# Evaluating the Instructional Design and Effect on Knowledge, Teamwork, and Skills of Technology-Enhanced Simulation-Based Training in Obstetrics in Uganda: Stepped-Wedge Cluster Randomized Trial

**DOI:** 10.2196/17277

**Published:** 2021-02-05

**Authors:** Anne Antonia Cornelia van Tetering, Maartje Henrica Martine Segers, Peter Ntuyo, Imelda Namagambe, M Beatrijs van der Hout-van der Jagt, Josaphat K Byamugisha, S Guid Oei

**Affiliations:** 1 Department of Obstetrics and Gynecology Máxima Medical Center Veldhoven Netherlands; 2 Department of Obstetrics and Gynecology Mulago Hospital Makerere University College of Health Sciences Kampala Uganda; 3 Department of Electrical Engineering Eindhoven University of Technology Eindhoven Netherlands; 4 Department of Biomedical Engineering Eindhoven University of Technology Eindhoven Netherlands

**Keywords:** simulation training, medical education, instructional design, low- and middle-income countries, obstetrics

## Abstract

**Background:**

Simulation-based training is a common strategy for improving the quality of facility-based maternity services and is often evaluated using Kirkpatrick’s theoretical model. The results on the Kirkpatrick levels are closely related to the quality of the instructional design of a training program. The instructional design is generally defined as the “set of prescriptions for teaching methods to improve the quality of instruction with a goal of optimizing learning outcomes.”

**Objective:**

The aim of this study is to evaluate the instructional design of a technology-enhanced simulation-based training in obstetrics, the reaction of participants, and the effect on knowledge, teamwork, and skills in a low-income country.

**Methods:**

A stepped-wedge cluster randomized trial was performed in a university hospital in Kampala, Uganda, with an annual delivery volume of over 31,000. In November 2014, a medical simulation center was installed with a full-body birthing simulator (Noelle S550, Gaumard Scientific), an interactive neonate (Simon S102 Newborn CPR Simulator, Gaumard Scientific), and an audio and video recording system. Twelve local obstetricians were trained and certified as medical simulation trainers. From 2014 to 2016, training was provided to 57 residents in groups of 6 to 9 students. Descriptive statistics were calculated for ten instructional design features of the training course measured by the 42-item ID-SIM (Instructional Design of a Simulation Improved by Monitoring). The Wilcoxon signed rank test was conducted to investigate the differences in scores on knowledge, the Clinical Teamwork Scale, and medical technical skills.

**Results:**

The mean scores on the ten instructional design features ranged from 54.9 (95% CI 48.5-61.3) to 84.3 (95% CI 80.9-87.6) out of 100. The highest mean score was given on the feature feedback and the lowest scores on *repetitive practice* and *controlled environment*. The overall score for the training day was 92.8 out of 100 (95% CI 89.5-96.1). Knowledge improved significantly, with a test score of 63.4% (95% CI 60.7-66.1) before and 78.9% (95% CI 76.8-81.1) after the training (*P*<.001). The overall score on the 10-point Clinical Teamwork Scale was 6.0 (95% CI 4.4-7.6) before and 5.9 (95% CI 4.5-7.2) after the training (*P*=.78). Medical technical skills were scored at 55.5% (95% CI 47.2-63.8) before and 65.6% (95% CI 56.5-74.7) after training (*P*=.08).

**Conclusions:**

Most instructional design features of a technology-enhanced simulation-based training in obstetrics in a low-income country were scored high, although intervals were large. The overall score for the training day was high, and knowledge did improve after the training program, but no changes in teamwork and (most) medical technical skills were found. The lowest-scored instructional design features may be improved to achieve further learning aims.

**Trial Registration:**

ISRCTN Registry ISRCTN98617255; http://www.isrctn.com/ISRCTN98617255

**International Registered Report Identifier (IRRID):**

RR2-10.1186/s12884-020-03050-3

## Introduction

### Maternity Care

The improvement of maternal and newborn care is a global priority. The United Nations constructed the Millennium Development Goals and the Sustainable Development Goals, in which the aim of reducing the maternal and neonatal mortality was included [1]. Targets for 2030 are to reduce the global maternal mortality ratio to less than 70 per 100,000 live births and to reduce neonatal mortality to at least as low as 12 per 1000 live births [1]. In Uganda, in 2015 the maternal mortality ratio was still 343 per 100,000 live births, and the neonatal mortality rate was 20.2 per 1000 live births in 2017 [2,3]. Shortage of trained staff, poor management of emergency obstetric care provision, poor referral practices, and poor coordination among staff are barriers that hinder or delay the ability to access emergency obstetric services [4]. Simulation-based medical team training may have a positive effect on these barriers.

### Simulation-Based Training

Simulation-based training in low-income and middle-income countries usually focuses on improving capacity and providing safe clinical skills to directly reduce maternal and neonatal mortality and morbidity [5]. A review in 2010 about training programs in low-resource environments aimed at improving emergency obstetric care concluded that training programs may improve quality of care, but strong evidence was lacking [6]. Since this review, there have been numerous evaluation studies on the effectiveness of simulation training for obstetric emergencies in low-income and middle-income countries [7-40]. The results of these studies show that obstetric simulation training is associated with improvements in clinical outcomes, mostly neonatal outcomes [7,11,16,18,24,26,28,33,36,38,40]. A later review included 23 studies about the impact of multiprofessional emergency obstetric and neonatal care training in high-income, middle-income, and low-income countries [5]. The conclusion of this review was that this type of training does make a difference [5]. Progress was not only found with regard to individual knowledge, skills, and attitudes, but also with regard to longer-term change in behavior and improvements in maternal and neonatal morbidity and mortality [5]. Sufficient evidence exists to justify the expense and effort of it [5]. Draycott et al agreed with this, but also mentioned that not all training is clinically effective and results are not entirely consistent [41]. Further research on the evaluation of different training programs is necessary to understand why some training programs improve clinical outcomes, and others show no improvements or even deterioration in outcomes.

### Evaluating Simulation-Based Training

Most evaluation studies on simulation-based training in low-income and middle-income countries used Kirkpatrick’s theoretical model. This model is composed of four levels: reaction, learning, behavior, and results. Each successive level of the model represents a more precise measure of the effectiveness of a training program. The results on these Kirkpatrick levels are closely related to the quality of the instructional design of a training program [42]. The instructional design is generally defined as the “set of prescriptions for teaching methods to improve the quality of instruction with a goal of optimizing learning outcomes” [43]. Another name for these prescriptions is *affordances* with the purpose of maximizing the effect, effectiveness, and usefulness of an educational instrument [44]. The instructional design of the training program may influence the outcomes on the Kirkpatrick levels [45]. Therefore, if the learning aim is not met, this may have to do with an inappropriate design.

A review on postgraduate medical e-learning recommended not only to evaluate the outcomes of an educational intervention, but to start with evaluation of its design [45]. For simulation-based medical education, Issenberg et al and McGaghie et al have described essential instructional design features [42,46]. These include feedback, repetitive practice, ranging difficulty levels, defined outcomes, individualized learning, curriculum integration, multiple learning strategies, clinical variation, controlled environment, and simulator validity [42,46]. These features were integrated by Fransen et al in the ID-SIM (Instructional Design of a Simulation Improved by Monitoring), an evidence-based assessment tool that can be used to aid development and evaluation of the instructional design of a simulation-based team training [47].

### Training for Life

A technology-enhanced simulation-based training in emergency obstetrics was developed in Mulago Hospital in Kampala, Uganda (Training for Life). The training focused on both medical technical skills and teamwork. To evaluate the training program, we conducted a stepped-wedge cluster randomized trial. In this paper, we present the results of the evaluation of the instructional design of this training program, the reaction of participants, and the effect on knowledge, teamwork, and medical technical skills (Kirkpatrick levels 1 and 2).

## Methods

### Recruitment

Between October 2014 and April 2016, a stepped-wedge cluster randomized trial was conducted to implement technology-enhanced simulation-based team training in obstetrics. This educational intervention took place at the Makerere University College of Health Sciences, situated in Mulago Hospital in Kampala, Uganda. In November 2014, a medical simulation center was installed with a full-body birthing simulator (Noelle S550, Gaumard Scientific), an interactive neonate (S102 Simon Newborn CPR Simulator, Gaumard Scientific), and an audio and video recording system. Mulago Hospital is a national referral hospital in Kampala with an annual delivery volume of approximately 31,000. Over 23,000 women deliver at a medium-to-high–risk ward, and the staff of this ward consists of 45 gynecologists, 60 residents (first-year, second-year, and third-year senior house officers [SHOs]), and 45 midwives. To be included in the study, SHOs had to work at the medium-to-high–risk maternity ward of Mulago Hospital. As this study was set up as a stepped-wedge cluster randomized trial, clusters of SHOs started in a control period. Therefore, recruitment was done before the official opening of the simulation center and the train-the-trainers course. Seven clusters of first-year, second-year, and third-year SHOs were randomly created by a scheduler. To evaluate clinical outcomes, the SHOs had to work in the hospital in these fixed clusters during the study period.

Training for Life used a train-the-trainer model in which training was cascaded down from master trainers to local facilitators to learners. The group of master trainers consisted of two Dutch obstetricians, one communication expert, and one simulation specialist. They were all certified simulation educators. Twelve local senior obstetricians finalized a four-day training program and were certified as facilitators. Course materials were developed in cooperation with staff members in Mulago Hospital and Medsim, a medical simulation center in Eindhoven, the Netherlands. All materials were provided in English.

After the train-the-trainers course, training was cascaded down to the SHOs. Each training was given by two recently certified local facilitators to 7 clusters of each 6 to 9 SHOs of different study years. The training comprised a one-day (8-hour) simulation-based acute obstetric training focusing on medical technical skills and teamwork/crew resource management (eg, closed-loop communication, leadership, speaking up). The two facilitators focused alternately on medical technical skills or crew resource management. Scenarios included postpartum hemorrhage, eclampsia, ventouse delivery followed by resuscitation of the newborn, breech delivery, and a repetition of postpartum hemorrhage with a different etiological mechanism. Every scenario was briefly introduced by the medical facilitator, and after each scenario, a debriefing with review of the video recordings was provided with feedback on medical technical skills and crew resource management. All scenarios were performed once, according to a fixed script with realistic clinical progress. At least three SHOs could participate actively in each scenario. After the main training, at least one half-day repetition training session was organized for each group.

As this study was set up as a stepped-wedge cluster randomized trial, all 7 clusters of SHOs started in the control condition. Then, all clusters received the training at consecutive time points, scheduled 7 weeks apart. The order of the switch per cluster was randomized by a computer. Eventually, all clusters switched from the control to the training condition.

### Instructional Design

This study evaluates the instructional design of the training and the effect of the training on Kirkpatrick levels 1 and 2. The instructional design was measured using the ID-SIM [47]. This questionnaire is an assessment tool, specifically designed for the evaluation of the instructional design of a simulation-based team training [47]. It consists of 42 statements that can be answered by placing a mark on a line from “not at all/never” to “completely/always”. The questions are divided over ten instructional design features: feedback, repetitive practice, curriculum integration, difficulty range, learning strategies, clinical variation, controlled environment, defined outcomes, individualized learning, and simulation fidelity.

### Kirkpatrick Levels 1 and 2

Kirkpatrick level 1 was measured by asking all participants to give an overall score for the training day by placing a mark on a line. Suggestions for improvement could be made in an open remark at the end of the evaluation questionnaire. Level 2, the effect on knowledge of the participants, was measured by a knowledge test consisting of 30 multiple-choice questions on medical technical skills and teamwork at the beginning and end of the main training (Multimedia Appendix 1). To obtain content validity, a team of Dutch and Ugandan obstetricians developed and evaluated the multiple-choice questions. Construct validity was tested by asking obstetricians and first-year, second-year, and third-year SHOs to complete the knowledge test. A Cronbach α coefficient was calculated to measure the internal consistency of the knowledge test.

The effect on technical skills and teamwork was evaluated by assessing the video-recorded scenarios. Three independent researchers assessed the first and last scenario for medical technical skills and teamwork together until consensus was reached. The topic of both scenarios was postpartum hemorrhage; however, the etiology differed. The assessors were blinded for the day of training and whether the scenario was the first or the last of the day. The assessment consisted of the Clinical Teamwork Scale (CTS) and a checklist of medical technical procedures. The CTS is a validated tool for assessing teamwork [48]. It consists of 15 items about communication, situational awareness, decision-making, and role responsibility, and each can be scored on a 10-point scale. The checklist of medical technical procedures is based on local protocols for postpartum hemorrhage, and it consists of 24 items that can be either scored as “done,” “not done,” or “not applicable.”

### Statistical Analysis

This paper shows secondary outcome results. A sample size calculation was performed based on the primary outcome of the study (the combined mortality proportion including maternal and neonatal mortality ratios). For a stepped-wedge design, first the sample size calculation for a standard randomized clinical trial is required [49-51]. To show a reduction in combined mortality proportion of 20% with an α of .05 and a power of 80%, a total of 6398 deliveries were needed for a standard randomized clinical trial design. The design effect was then calculated assuming an intracluster correlation of 0.05, 7 clusters, and a cluster size of 3343 deliveries per year, which resulted in 2367 deliveries per cluster period. This resulted in a minimum duration of 5 weeks for each cluster period based on local delivery rates. For logistical reasons in staff scheduling, the duration of each step was set at 7 weeks. As exam and holiday periods were excluded from the cluster periods, the total duration of the study was anticipated to be 1.5 years. Data were analyzed using IBM SPSS Statistics, version 21 (IBM Corporation). Descriptive statistics were calculated for participant characteristics and for the results of the ID-SIM. The Wilcoxon signed rank test was conducted to investigate the difference in scores on the knowledge test, the CTS, and medical technical skills assessment. The difference in scores on the knowledge test between the SHOs in their first, second, and third years of study was analyzed using the Kruskal-Wallis test. Statistical significance was accepted at a 2-sided *P* value of .05.

### Ethical Permission

Ethical permission was obtained from both the Mulago Research and Ethics committee (Protocol MREC: 674) and the Uganda National Council for Science and Technology (number SS 3927). All participants gave written informed consent before the study began, and they acknowledged that they cannot be identified via the paper. Data were fully anonymized.

## Results

### Learner Characteristics

From 2014 to 2016, 68 SHOs were invited to participate in the training program; 19 (28%) of them were female, and 49 (72%) were male. Of these, 57 SHOs (84%) participated in the main training, with an even distribution over the three years of their obstetric curriculum (20 first-year SHOs, 18 second-year SHOs, and 19 third-year SHOs). Of the 11 SHOs who did not participate in the main training, 3 finalized their specialization, 1 quit specialization, and 7 did not give any reason. Almost half of the SHOs (49%, 33/68) took part in at least one repetition training. The total number of trained SHOs was higher than the average working number, because of the organization of extra main training sessions for leaving SHOs and the new first-year SHOs who were added to an already trained cluster.

### Instructional Design

All of the 57 SHOs who participated in the main training completed the ID-SIM. The mean scores of the ten instructional design features are shown in Table 1. Mean scores on the features differed between 54.9 and 84.3 out of 100. The highest mean score of 84.3 (95% CI 80.9-87.6) was given on *feedback*. The lowest scores of 62.8 (95% CI 55.8-69.8) and 54.9 (95% CI 48.5-61.3) were given on *repetitive practice* and *controlled environment*, respectively.

**Table 1:** Mean scores of senior house officers on the ID-SIM.

Variable	ID-SIM score, mean (95% CI)
Feedback	84.3 (80.9-87.6)
Repetitive practice	62.8 (55.8-69.8)
Curriculum integration	78.7 (74.5-82.9)
Difficulty range	74.0 (68.5-79.4)
Learning strategies	83.2 (78.9-87.4)
Clinical variation	80.0 (74.9-85.1)
Controlled environment	54.9 (48.5-61.3)
Individualized learning	81.9 (76.9-86.9)
Defined outcomes	74.2 (69.2-79.3)
Simulation fidelity	80.3 (76.9-83.7)

### Kirkpatrick Levels 1 and 2

The overall score for the training day rated by the participants was 92.8 out of 100 (95% CI 89.5-96.1). The following suggestions for improvement were made in the open remark at the end of the questionnaire: (1) to incorporate other members of the team, (2) to add other scenarios, (3) to have repetition training more often, (4) to plan more time for the debriefing, especially relating to a real-life setting, and (5) to provide the training materials a day earlier.

Of the 57 participating SHOs, a total of 53 (93%) completed the knowledge test before and after the main training. One SHO completed the knowledge test only after the training. Construct validity was tested using the Kruskal-Wallis test to compare knowledge test results of obstetricians and first-year, second-year, and third-year SHOs and showed a significant result (*P*=.03). A Cronbach α coefficient of .67 was calculated to measure the internal consistency of the knowledge test. Mean scores of the knowledge test are listed in Table 2. The mean score of the knowledge test increased from the beginning to the end of the training day. This result was also found for each study year separately. The improvement in score on the knowledge test between the three study years was not significantly different (*P*=.24).

**Table 2:** Mean scores of senior house officers on the knowledge test.

Year of study	Score before training, mean (95% CI)	Score after training, mean (95% CI)	*P* value
All	63.4 (60.7-66.1)	78.9 (76.8-81.1)	<.001
1st year	62.3 (58.3-66.4)	77.7 (72.5-82.8)	<.001
2nd year	60.9 (56.1-65.7)	78.9 (76.7-81.1)	<.001
3rd year	68.1 (62.5-73.7)	80.7 (77.2-84.1)	.001

To evaluate teamwork and medical technical skills, the recordings of the first and last scenarios of 8 teams were evaluated. Out of 16 recordings, 2 could not be assessed because of recording issues. No differences in scores on the CTS between the first and last sessions were found (Table 3). The scores of the technical skills assessment only improved statistically significantly for the provision of drugs (Table 3). During the first scenario, none of the teams reached the moment to tamponade the uterus. For 5 out of the 8 teams, the last scenario was stopped before they had to tamponade the uterus, hence this item was scored as not applicable. The scenarios were stopped by the local facilitators at the moment when they judged that the SHOs had reached sufficient learning subjects to discuss in the debriefing sessions.

**Table 3:** Mean scores of senior house officers in clusters on the Clinical Teamwork Scale and the medical technical skills assessment.

Item		First scenario score, mean (95% CI)	Fifth scenario score, mean (95% CI)	*P* value
**Clinical Teamwork Scale**				
	Overall score	6.0 (4.4-7.6)	5.9 (4.5-7.2)	.78
	Overall communication	6.5 (5.5-7.6)	6.0 (4.5-7.5)	.4
	Overall situational awareness	4.4 (2.8-6.0)	5.4 (4.5-6.2)	.1
	Overall decision making	4.6 (3.4-5.7)	6.0 (5.1-6.9)	.07
	Overall responsibility	6.6 (5.6-7.7)	6.0 (5.3-6.8)	.59
	Patient friendliness	5.6 (4.1-7.1)	6.0 (4.8-7.2)	.79
**Medical technical skills**				
	Overall score	55.5 (47.2-63.8)	65.6 (56.5-74.7)	.08
	Ask for help	100	100	>.99
	Airway, breathing, circulation	58.9 (45.9-72.0)	54.6 (43.0-66.2)	.89
	Establish cause	50.0 (25.2-74.8)	76.2 (41.9-110.5)	.34
	Massage uterus	57.1 (18.5-95.8)	66.7 (31.1-102.3)	.59
	Provision of drugs	28.6 (12.6-44.5)	56.0 (46.3-65.6)	.04
	Shift to theatre	85.7 (63.2-108.3)	78.6 (53.9-103.3)	.56
	Tamponade	N/A^a^	N/A	N/A

^a^N/A: not applicable.

## Discussion

### Principal Results

In this article, we investigated the instructional design of a technology-enhanced simulation-based training in obstetrics, the reaction of participants, and the effect on knowledge, teamwork, and medical technical skills of SHOs. Most instructional design features were scored high, although intervals were large. The highest-rated instructional design feature was *feedback,* and the lowest-rated were *repetitive practice* and *controlled environment*. The overall rating of the SHOs for the training program was high, with a mean score of 92.8 out of 100. Knowledge did increase after the training program, but no changes in teamwork and (most) technical skills were found. Results of the ID-SIM showed suggestions for improvement of the instructional design of the training program to achieve learning aims.

### Strengths and Limitations

This study evaluates both the instructional design of a technology-enhanced simulation-based training in obstetrics and the effect on Kirkpatrick levels 1 and 2 in a low-income country at one of the biggest maternity wards in the world. The validated ID-SIM was used to evaluate the instructional design of the training program. A limitation of the study may be that the ID-SIM was scored by the SHOs, who may not have much expertise in evaluating an instructional design. However, Fransen et al mentioned that the ID-SIM may be helpful for less-experienced individuals who are challenged with the development or evaluation of a simulation-based team training course [47]. Nevertheless, validation of participants’ ratings, instead of expert opinion, on the ID-SIM could be an item of further research.

Another limitation of this study is the level of expertise and the composition of the training groups. SHOs of different study years were divided into groups with a different team leader in the first and last scenario of the day. This means that the level of knowledge, skills, and teamwork of the team leader can differ between sessions. Other limitations include the ratio of male to female participants with 72% male participants, and missing data due to the dropout of 7 of the 68 SHOs without known reason, 4 SHOs who didn’t fill in the knowledge test, and 2 missing video recordings due to technical issues. Moreover, only 33 SHOs participated in at least one repetition training. Information on motivation and reasons for not participating in further training sessions should be included in further evaluation studies to optimize learning results. Furthermore, it was hard to specifically define the level of knowledge, teamwork, and medical technical skills in advance. This may have resulted in learning objectives that were not challenging enough for all SHOs. Additionally, the item *tamponade the uterus* in the medical technical skills could not be scored in the way it was originally planned, as most scenarios were stopped before the clusters reached the moment to practice this skill. Hence, evaluation on Kirkpatrick levels 3 and 4 will probably not show any effect of this training subjective. Finally, the training teams only consist of SHOs, as it was not feasible to create working schedules with fixed teams including midwives, interns, SHOs, obstetricians, anesthesiologists, and pediatricians. To measure the effects of the training program using a stepped-wedge cluster randomized trial in one hospital, fixed teams were necessary. As the SHOs are the first responders after the midwives in emergency care at the labor ward, we chose to focus on these care providers. However, we are aware that teamwork is critical to provide safe obstetric care. All of the previous studies that have reported improvements after training have implemented “in-house” training programs and have trained almost 100% of their staff [52]. These features seem to be two of the active components of effective training [52]. For future training, a multiprofessional training program is recommended.

### Comparison With Prior Work

De Leeuw et al have identified and compared the outcomes and methods used to evaluate postgraduate medical e-learning, including simulation [45]. Of the theories, Kirkpatrick’s hierarchy was the most used method [45]. However, many other ways to carry out an evaluation were found, and it is probable that many ways to do so are correct [45]. A recommendation by De Leeuw et al was to evaluate not only the outcomes of an educational intervention but to start with the evaluation of its design [45]. Robust instructional design is required to achieve an effective training course. Moreover, to perform comparisons between simulation-based team training courses, Eppich et al recommended standardized reporting of these instructional designs [42,53]. Issenberg et al translated the literature into ten important design features [46]. Five out of these ten features corresponded to the educational theory of deliberate practice by Ericsson et al [54,55]. Cook et al confirmed the effectiveness of several of Issenberg’s instructional design features [46,56]. The features were incorporated into two guidelines for designing an effective simulation-based training by the Association for Medical Education in Europe [57,58]. Later, Fransen et al developed, based on previous findings, an evidence-based assessment tool for evaluation of the instructional design of a simulation-based team training: the ID-SIM [47]. Table 1 shows the instructional design features of the technology-enhanced simulation-based training in obstetrics evaluated in this study. The table identifies the weaknesses in the instructional design of this training: *repetitive practice* and *controlled environment.*

### Repetitive Practice

There is increasing evidence of the beneficial effect of repetitive practice. Cook et al analyzed over 600 studies in a systematic review and meta-analysis and reported that the distribution of learning activities over more than one day was consistently associated with larger effect sizes [59]. Bluestone et al also described that repetitive, time-spaced education exposure resulted in better knowledge outcomes, better knowledge retention, and better clinical decisions compared with single interventions and live instruction [60]. Additionally, improvement in skills was demonstrated after various types of refresher courses [61-64]. A study from van de Ven et al reported that the beneficial effect of a one-day, simulation-based, multiprofessional obstetric team training seems to decline after 3 months [65]. Repetitive training sessions every 3 months are therefore recommended. However, in low-income and middle-income countries conflict may arise because having adequate time and support for simulation-based training can be a challenge. Several studies describe challenges of pulling staff both as learners and educators out of their workplaces because of staff shortages or complex schedules [14,17,66,67]. In particular, longer courses have struggled with high on-site dropout rates because of night call schedules [67]. More research is necessary to determine the optimal training intervals in low-income and middle-income countries. The effects of training programs with different intervals between repetition sessions on the four Kirkpatrick levels, but also on participants' dropout rates and participants' and trainers' motivation, should be investigated in order to optimize this instructional design feature in low-income and middle-income countries.

### Controlled Environment

The other lower-scored item on the ID-SIM was *controlled environment*. In a controlled clinical environment, learners can make, detect, and correct errors in patient care without adverse consequences. Moreover, instructors can focus on learners instead of patients. The low score in this study on this item may have to do with staff shortages and complex schedules. Training sessions were frequently interrupted by phone calls. Interference with clinical obligations may be a bigger issue in low-income and middle-income countries compared with high-income countries due to a shortage of personnel. Moreover, the educational system of Uganda differs from the system in high-income countries. In low-income to middle-income countries, health professionals may not be as familiar with simulation-based education as in high-income countries [68,69]. Moran et al even described the educators' lack of comfort with leading simulations as one of the key challenges in simulation-based training [69]. To increase the effectiveness of the training program*,* the *controlled environment* has to be improved*.*

### Conclusions

Most instructional design features of a technology-enhanced simulation-based training in obstetrics in a low-income country were scored high, although intervals were large. The highest mean score was given on *feedback*, and the lowest scores on *repetitive practice* and *controlled environment.* The overall score for the training day was high, and knowledge did improve after the training program, but no changes in teamwork and (most) medical technical skills were found. The lowest-scored instructional design features, *controlled environment* and *repetitive practice*, may be improved to achieve further learning aims. Future studies should also include evaluation of the instructional design of a training program in order to understand why some training programs are effective and others are not.

.

## Appendix

Multimedia Appendix 1 Knowledge test.

Multimedia Appendix 2 CONSORT checklist.

## Abbreviations

CTS: Clinical Teamwork Scale
ID-SIM: Instructional Design of a Simulation Improved by Monitoring
SHO: senior house officer
