# Supplementary material for: Evaluating the Instructional Design and Effect on Knowledge, Teamwork, and Skills of Technology-Enhanced Simulation-Based Training in Obstetrics in Uganda: Stepped-Wedge Cluster Randomized Trial
Source: JMIR Med Educ. 2021 Feb 5;7(1):e17277. doi: 10.2196/17277 (PMC8081249; doi:10.2196/17277)
Supplement: Multimedia Appendix 1 [file mededu_v7i1e17277_app1.docx]

Knowledge test

Select the best answer to each question or statement (always one answer)
Circle the letter of the correct answer.

**Name:**

**Time:**

1. **Crew resource management principles are**
2. Technical skills, leadership, prioritizing, and communication
3. Using flowcharts, prioritizing, teamwork, and communication
4. Situational awareness, leadership, teamwork, and communication
5. Time-out, using flowcharts, teamwork, and communication
6. **The target of millennium development goal four is**
7. To reduce under-five child mortality rate by two thirds between 1990 and 2015
8. To reduce perinatal child mortality rate by two thirds between 1990 and 2015
9. To reduce maternal mortality rate by three quarters between 1990 and 2015
10. To integrate the principles of [sustainable development](http://en.wikipedia.org/wiki/Sustainable_development) into country policies and programs
11. **The most common cause of maternal mortality in New Mulago hospital in 2013 was**

a. Puerperal sepsis

b. Abortion

c. Eclampsia

d. Thrombosis

1. **An example of the S in SBAR is**

a. “Miss R has a postpartum haemorrhage”

b. “Miss R has a low BP, high pulse and we have given 1L of NaCl”

c. “Miss R is a G4P2 and had a fresh still birth in her previous pregnancy”

d. “When shall I call you again?”

1. **Which answer is not true.**

**During a time-out you have to ask yourself...**

1. What has been done already?
2. What has to be done?
3. What is the new plan?
4. What did I do wrong?
5. **'Can you give 10IU Oxytocin?' 'Yes, I will give 10IU Oxytocin.' 'I have given 10IU Oxytocin.'**

**The last sentence of this example is...**

a. The R in SBAR

b. Closed loop communication

c. Ten to ten moment

d. Good leadership

1. **The A in SBAR means**

a. Acknowledgement

b. Assessment

c. Appointment

d. Appraisal

1. **Closed loop communication means**

a. The manner in which a team member makes it clear to the team members which extra materials are needed in an acute situation

b. The manner in which a team member makes it clear to another team member to speak up

c. The manner in which a team member makes it clear to another team member which person they should call in an acute situation.

d. The manner in which a team member makes it clear to another team member that the requested task is performed, by means of repetition and confirmation

1. **An example of the R in SBAR is**

a. I call for this patient...

b. Medical history....

c. I want you to come over....

d. Blood pressure is 110/55...

1. **What is not true. You prevent infections if...**

a. You wash your hands after every contact with patients

b. You wear long gloves during manual placenta removal

c. You use sterile materials during surgery

d. You wash your hands once at the beginning of the day

1. **Which of the following lists contains the three MAIN causes of bleeding after birth?**

a. Soft uterus, breech, retained placenta

b. Soft uterus, a retained placenta, and perineal tearing

c. Soft uterus, twins, and dehydration

d. Retained placenta, malaria, and perineal tearing

1. **The M in acronym HAEMOSTASIS means**

a. Manipulate the uterus

b. Massage the uterus

c. Monitor the uterus

d. Manual removal

1. **When a mother starts heavily bleeding after delivery, I have to**

a. Call for help, airway/breathing/circulation, establish cause

b. Call for help, airway/breathing/circulation, massage uterus

c. Establish cause, call for help, airway/breathing/circulation

d. Airway/breathing/circulation, call for help, establish cause

1. **The definition of postpartum haemorrhage after spontaneous vaginal delivery**

a. Blood los more than 1000 ml

b. Blood loss more than 500ml

c. Blood loss more than 750 ml

d. Blood loss more than 1250 ml

1. **The last resort to solve postpartum haemorrhage is to**

a. Apply Bakri balloon

b. Apply B-lynch

c. Perform Supravaginal hysterectomy

d. Perform Supra-abdominal hysterectomy

1. **Treat eclampsia with**

a. Magnesium sulphate 4 grams i.v. over 20 minutes

b. Magnesium sulphate 40 grams i.v. over 20 minutes

c. Diazepam 50 mg i.v.

d. Diazepam 5 mg i.v.

1. **Complications of preeclampsia**

a. Haemolysis and liver rupture

b. Pneumonia and acute tubules necrosis

c. Diffuse intravascular coagulation and sepsis

d. Anaemia and pulmonary embolism

1. **How many of the eclamptic insults occur postpartum**

a. 2%

b. 15%

c. 44%

d. 66%

1. **Control blood pressure with antihypertensive medication if diastolic blood pressure raises above the level of**

a. 110

b. 80

c. 160

d. 140

1. **First choice medication to decrease blood pressure after/during eclampsia and DBP >110 mmHg**

a. Hydralizine 5 mg IV q 30 min

b. MgSO_4_ 4 g IV over 20 min

c. Ergometrine 0.4 mg IM

d. Labetalol 20 mg q 60 min

1. **Neonatal resuscitation ratio chest compressions to insufflation breaths**

a. 3:1

b. 30:2

c. 15:2

d. 4:1

1. **Neonatal resuscitation rhythm of chest compressions**

a. 50/min

b. 100/min

c. 120/min

d. 150/min

1. **
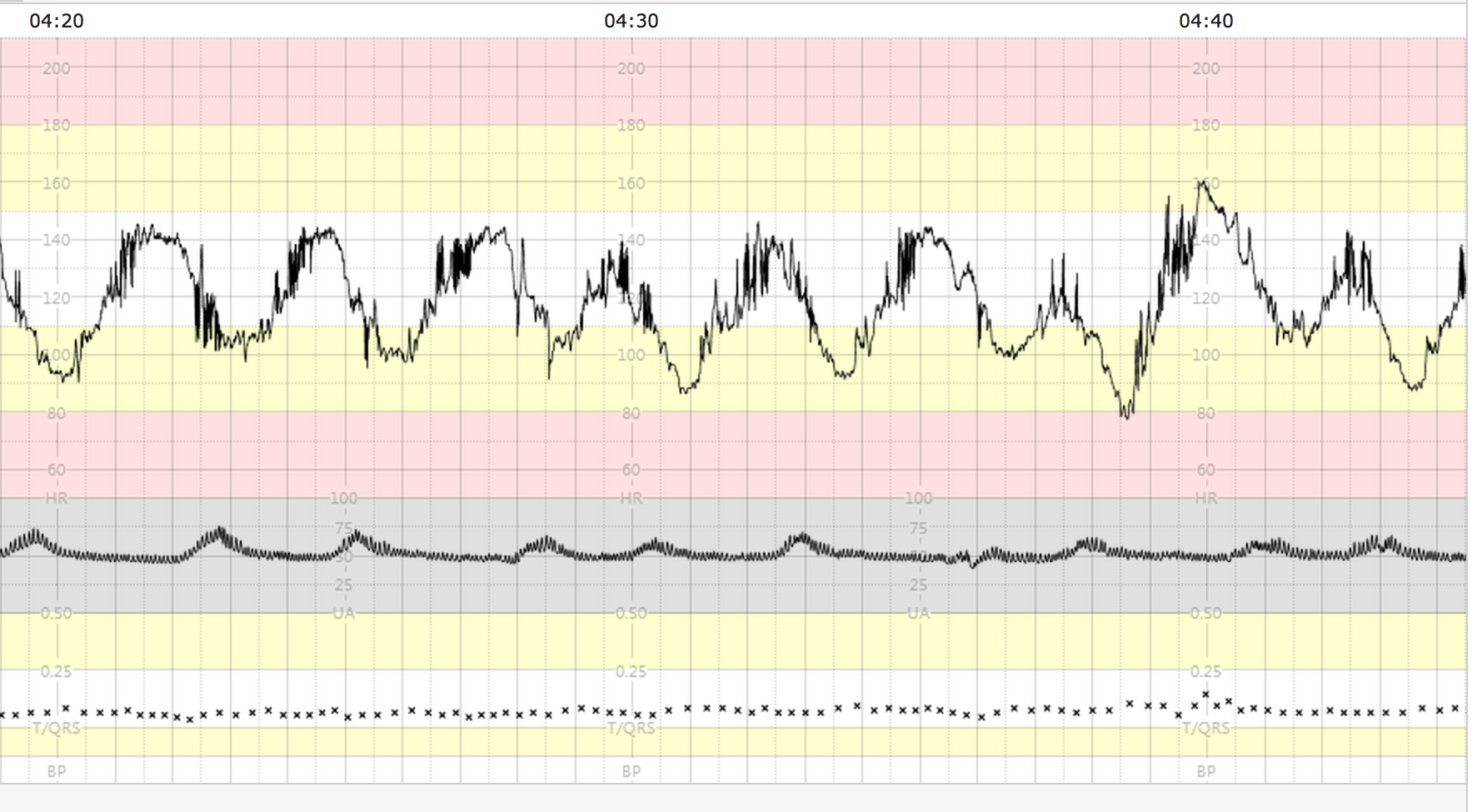
Give your conclusion about this CTG:**

a. Optimal

b. Preterminal

c. Suboptimal

d. Abnormal

1. **NOT an indication for vacuum extraction is**

a. Maternal exhaustion

b. Fetal distress in the second stage of labour

c. Need to shorten the second stage of labour for maternal medical condition

d. Incompletely dilated cervix

1. **An absolute contraindication for a vacuum extraction is**

a. Breech, face or brow presentation

b. Not motivated patient

c. Gestation 34-37 weeks

d. Intrauterine grow restriction

1. **Risk factors for a breech delivery are**

a. Multiparity

b. History of breech delivery

c. Obesity

d. Intrauterine grow restriction

1. **During a breech delivery, when would you grab the neonate?**

a. As soon as you see the legs

b. When you see the buttock of the baby

c. When you see the scapula

d. After delivery of the total chest

1. **During a breech delivery, when the head is not born during spontaneous breech delivery, I should use...**

a. Løvset’s manoeuvre


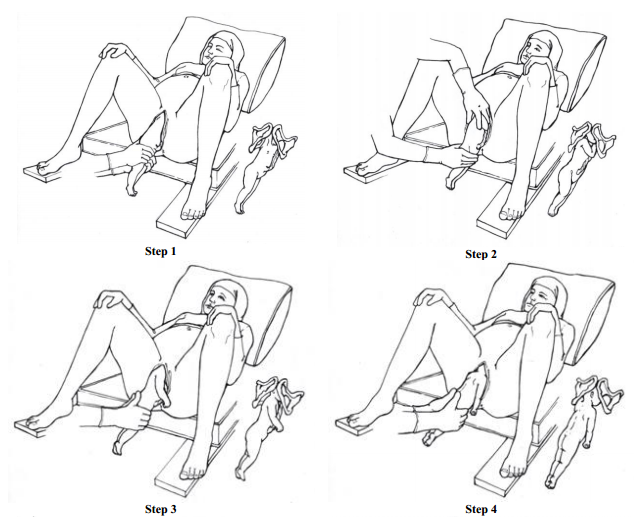
b. Classical method

c. Mauriceau manoeuvre

d. A vacuum extraction

**29. These steps on the right side of the page (picture) are called:**

a. Løvset’s manoeuvre

b. Classical method

c. Mauriceau manoeuvre

d. All fours

**30. During a breech delivery on all fours position:**

a. Back of the baby turns towards the abdomen of the mother

b. Back of the baby turns towards the back of the mother

c. The back of the baby will go upwards

d. The face of the baby won't look at you

Thank you!
